# Can Leukotriene Receptor Antagonist Therapy Improve the Control of Patients with Severe Asthma on Biological Therapy and Coexisting Bronchiectasis? A Pilot Study

**DOI:** 10.3390/jcm11164702

**Published:** 2022-08-11

**Authors:** Vitaliano Nicola Quaranta, Silvano Dragonieri, Nunzio Crimi, Claudia Crimi, Pierachille Santus, Francesco Menzella, Corrado Pelaia, Giulia Scioscia, Cristiano Caruso, Elena Bargagli, Konstantinos Kostikas, Christos Kyriakopoulos, Nicola Scichilone, Giovanna Elisiana Carpagnano

**Affiliations:** 1 Department of Basic Medical Sciences, Neuroscience and Sense Organs, Section of Respiratory Disease, University “Aldo Moro” of Bari, 70121 Bari, Italy; 2 Department of Internal Medicine and Specialistic Medicine, Section of Respiratory Diseases, University of Catania, 95123 Catania, Italy; 3 Department of Clinical and Biomedical Sciences, Division of Respiratory Diseases, Università degli Studi di Milano, “L. Sacco” University Hospital, ASST Fatebenefratelli-Sacco, 20157 Milan, Italy; 4 Pulmonology Unit, S. Valentino Hospital, 31044 Montebelluna City, Italy; 5 Department of Health Sciences, Section of Respiratory Disease, University ‘‘Magna Græcia’’ of Catanzaro, 88100 Catanzaro, Italy; 6 Department of Medical and Surgical Sciences, University Hospital Policlinico Riuniti of Foggia, 71122 Foggia, Italy; 7 UOSD DH Gastroenterology Polyclinic Foundation A. Gemelli IRCCS, Department of Medical and Surgical Sciences, Cattolica University del Sacro Cuore, 00168 Rome, Italy; 8 Respiratory Diseases and Lung Transplant Unit, Department of Medical and Surgical Sciences and Neurosciences, University of Siena, 53100 Siena, Italy; 9 Respiratory Medicine Department, University of Ioannina School of Medicine, 45500 Ioannina, Greece; 10 Promise Department, University of Palermo, 90133 Palermo, Italy

**Keywords:** severe asthma, LTRA therapy, bronchiectasis, biological

## Abstract

Introduction: Asthma and bronchiectasis appear to be two related diseases and in their complex inflammatory interaction, the cysteinyl leukotriene/cysteinyl leukotriene receptor 1 (cysLT/cysLTR1) axis appears to play an important role given its involvement also in the neutrophilic pathway. To our knowledge, few studies have been conducted so far to evaluate the role of the leukotriene cysLT/cysLTr1 axis in the management of clinical and inflammatory outcomes within a population of patients with severe asthma and bronchiectasis. The aim of our study was to verify in this population the effect of leukotriene receptor antagonist (LTRA) therapy in clinical and inflammatory control before and after 6 months of introduction of biologic therapy. Methods: We retrospectively enrolled, from eight different severe asthma centers’ outpatients, 36 atopic patients with the simultaneous presence of non-cystic fibrosis (non-CF) and non-allergic bronchopulmonary aspergillosis (non-ABPA) bronchiectasis and severe asthma. The first biological injection was performed at baseline (T0 time). Patients who were already taking LTRA therapy at time T0 were recorded, and no new prescriptions were made. We observed our population over a 6-month period (T1 time). At the baseline we collected the following data: baseline characteristics, clinical history, high resolution computed tomography and bronchiectasis-related parameters and skin prick test. At both times T0 and T1 we collected the following data: asthma control test (ACT), asthma control questionnaire (ACQ), immunoglobulin E (IgE) level, blood count, fractional exhaled nitric oxide 50 (FeNO 50) and flow-volume spirometry. The study was retrospectively registered. Results: Our population had a mean age of 59.08 ± 11.09 and 50% were female. At T1, patients on LTRA therapy had a significantly lower FeNO value (33.03 ± 23.61 vs. 88.92 ± 77.96; *p* = 0.012). We assessed that the value of ΔFeNO (FeNO 50 T1 − FeNO 50 T0) and the number of unplanned specialist visits allowed a discrimination of 66.7% in the presence of LTRA therapy. We also verified how low FeNO values at time T1 were statistically significant predictors of LTRA therapy (ODD = 9.96 (0.94–0.99); *p* = 0.032). Conclusion: The presence of LTRA in therapy in a population of severe asthmatics with coexisting non-ASBPA bronchiectasis and non-cystic fibrosis, acting simultaneously on the T helper type 2 (TH2) pathway and probably on the neutrophilic component of bronchiectasis, would allow a further amplification of the beneficial effects of biological therapy, leading to a reduction in the number of unplanned visits to specialists.

## 1. Introduction

Asthma and bronchiectasis appear to be two related diseases, although the complexity of their relationship and mutual interaction are still a matter of debate. Both diseases are heterogeneous in terms of clinical symptoms and outcomes [1]. Interestingly, a number of studies have shown that the prevalence of bronchiectasis among patients with severe asthma ranges from 24% to 67.5% [2,3].

Regardless of a simple epidemiological association or a pathophysiological link, patients suffering from asthma and bronchiectasis may deserve a more specific therapeutic and follow-up management. Indeed, recent guidelines indicate that the presence of bronchiectasis should not modify asthma therapy [4,5,6]. On the contrary, the latest document of the Global Initiative for Asthma states that the primary goal for achieving control of asthmatic patients is based on identification and management of comorbidities, including bronchiectasis.

Asthma and bronchiectasis, as well as the neutrophilic and eosinophilic pathways, may have points of intersection [7,8]. It is likely that activated macrophages and neutrophils may express inducible nitric oxide synthase (iNOS) in bronchiectasis airways, since these cell types have been shown to express iNOS in response to cytokines. Thus, neutrophilic inflammation can produce nitric oxide NO in patients with bronchiectasis [9]. Furthermore, the cysteinyl leukotriene/cysteinyl leukotriene receptor 1 (cysLT/cysLTR1) axis is widely known in the immunopathogenesis of bronchial asthma as well as allergic rhinitis. However, the action of the cysLT/cysLTR1 axis is also expressed at the level of neutrophils [10]. Therapeutic management of the cysLT/cysLTR1 axis would therefore potentially control both the eosinophilic and neutrophilic pathways.

To our knowledge, no studies have been conducted so far to evaluate the role of the leukotriene cysLT/cysLTr1 axis in the management of clinical and inflammatory outcomes within a population of patients with severe asthma on biological therapy and non-CF (non-cystic fibrosis) and non-ABPA (non-allergic bronchopulmonary aspergillosis) bronchiectasis.

The aim of our study was to verify the effect of leukotriene receptor antagonist (LTRA) therapy in clinical and inflammatory control among a population of patients suffering from severe uncontrolled asthma with coexisting non-CF and non-ABPA clinically important bronchiectasis, before and after 6 months of introduction of biologic therapy.

Second, to verify its potential use in clinical follow-up, we aimed to compare eosinophilia and FeNO 50 in this setting of patients between baseline and after the introduction of biological therapy. 

## 2. Materials and Methods

### 2.1. Patients

After initial screening, we retrospectively enrolled 36 patients from [8] severe asthma centers (SA-centers) throughout the entire Italian territory (Bari, Foggia, Ferrara, Catania, Palermo, Napoli, Milano, Roma and Reggio Emilia).

Inclusion criteria of the study were: atopic patients with the simultaneous presence of non-CF and non-ASBA bronchiectasis documented by chest computed tomography (CT) and severe uncontrolled asthma in Global Initiative for Asthma (GINA) step 4–5 treatment [4].

Severe asthma was defined according to the most recent guidelines as “asthma which requires treatment with high dose inhaled corticosteroids (ICS) plus a second controller (and/or systemic corticosteroids) to prevent it from becoming ‘uncontrolled’ or which remains ‘uncontrolled’ despite this therapy” [4,11].

Bronchiectasis was defined as the presence of both permanent bronchial dilatation on CT scan and the clinical manifestations of cough, sputum production and/or recurrent respiratory infections [5].

Exclusion criteria were: bronchiectasis due to cystic fibrosis (CF) or allergic bronchopulmonary aspergillosis (ABPA), autoimmune diseases with pulmonary involvement, all other lung diseases that are different from those of the inclusion criteria, cardiologic comorbidities such as ischemic and/or arrhythmias and neurologic comorbidities such as stroke, epilepsy, degenerative pathologies and neuromuscular conditions.

All participants were eligible for biological therapy, according to the most recent guidelines [4]. 

This study was carried out according to the principles of the Declaration of Helsinki and was approved by the local Ethics Committee of the “Riuniti” Hospital of Foggia (Institutional Review Board approval number 17/CE/2014). It was retrospectively registered, and all recruited patients gave their written informed consent.

### 2.2. Study Design

It is a retrospective multicenter observational study. We observed our population over a 6-month period, as part of their routine care in their respective SA-centers. All centers received a database to be filled out with the clinical, diagnostic and therapeutic data of their patients at baseline (T0) and after 6 months of biological therapy (T1).

At baseline (T0), the following data were collected: patients clinical characteristics, detailed evaluation of bronchiectasis [12], presence of bilateral bronchiectasis and number of lung segments with bronchiectasis and detailed medical and pharmacological history of asthma. At time T0 other data were collected:

- Need of inhaled reliever medication, adherence to therapy.
- Time of asthmatic disease expressed in years.
- Skin prick test (SPT) was performed in accordance with the guidelines [13].
- In accordance with the guidelines [14], flow/volume (F/V) spirometry and plethysmography were performed. We measured and recorded the forced vital capacity (FVC) and the forced expiratory volume during the first second of the forced breath (FEV1) from the F/V Spirometry and the residual volume (RV) from the plethysmography. The best of three reproducible measurements was selected and expressed as a percentage of the predicted value.
- FeNO 50 was measured using an electrochemical analyzer according to ATS-ERS recommendations for online measurement of FeNO in adults [15,16].
- Questionnaires related to asthma disease were administered: asthma control test (ACT) [17], asthma control questionnaire (ACQ) [18].
- Questionnaires related to bronchiectasis were administered: bronchiectasis severity index (BSI) score [12], FACED (an acronym for FEV1, age, chronic pseudomonas aeruginosa bronchial infection colonization, radiological extension and dyspnea) score [19].
- A venous blood sample was taken, and white blood cell (WBC) values were recorded: WBCs, eosinophilia (EOS) and total immunoglobulin E (IgE).

In the last year prior to enrollment at T0, the following clinical outcomes were recorded:

- The number of courses of oral corticosteroid (OCS)/year, which is defined as the annual number of OCS prescriptions. The mean dose of OCS prescribed is reported as prednisone-equivalent dosages.
- The number of antibiotic courses/year, which is defined as the annual number of antibiotic prescriptions
- The number of bronchial exacerbations/year, which is defined as the presence of symptoms related to either an asthma exacerbation or a bronchiectasis exacerbation. Asthma exacerbations are defined as acute worsening in symptoms and lung function from the patient’s usual status [4]. Bronchiectasis exacerbations are defined by an increase in daily respiratory symptoms such as cough, sputum production, malaise, fatigue and breathlessness [5].
- The number of unscheduled specialist visits/year: number of urgent and unscheduled pulmonary visits without subsequent hospitalization in the last year.
- The number of hospitalizations/year: number of hospitalizations due to bronchial exacerbations caused by asthma and/or bronchiectasis in the last year.

The first biological injection (omalizumab, mepolizumab or benralizumab) was performed not later than 7 days from T0. Patients who at time T0 were already taking LTRA therapy were recorded. After 6 months from first injection (T1), patients returned to their SA-centers and repeated exactly the same procedures as at T0, except for bronchiectasis evaluation. At T1 time, the number of courses of oral corticosteroids (OCS), antibiotic courses, bronchial exacerbations, unscheduled specialist visits and hospitalizations were recorded during the 6 months of taking biological therapy.

We made a sub-analysis comparing the collected data of the 6 months pre-biological therapy with the data of the 6 months post-introduction of the therapy.

Subsequently, the study population was divided into 2 subgroups according to the trend of the FeNO 50 parameter between time T0 and time T1: Group 1, which showed a reduction in the FeNO 50 value from time T0 to time T1 (ΔFeNO 50 < 0) and Group 0, which did not show a reduction in the FeNO 50 value from time T0 to time T1 (ΔFeNO 50 ≥ 0). In the text we will use FeNO instead of FeNO 50.

FeNO 50 and eosinophilia are inexpensive and easy to measure in clinical practice [4]. FeNO 50, which has a known role in the clinical control of asthmatic patients [4], has also been seen to play an important role in bronchiectasis. Since FeNO production is also influenced by bronchiectasis [9], we wanted to study FeNO 50 in a subpopulation of patients suffering from severe uncontrolled asthma and coexisting non-CF and non-ABPA clinically important bronchiectasis to verify its potential use in clinical follow-up after the introduction of biological therapy.

### 2.3. Skin Prick Test

The skin prick test (SPT) was performed for a panel of inhalant allergens as previously described for common aeroallergens (Lofarma, Milan, Italy). It was considered positive when eliciting a wheal diameter ≥ 3 mm using negative (saline) and positive (histamine 10 mg/mL) controls for interpretation [13].

### 2.4. Lung Function

In accordance with the guidelines [14], FEV1 and FVC and RV were measured using a spirometer with body plethysmography (Jaeger, Essen, Germany). Respiratory tests were performed by experienced technicians under the supervision of a pneumologist. We measured and recorded the forced vital capacity (FVC) and the forced expiratory volume during the first second of the forced breath (FEV1) from the F/V spirometry and the residual volume (RV) from the plethysmography. The best of three reproducible measurements was selected and expressed as a percentage of the predicted value.

### 2.5. FeNO Measurement

FeNO was measured using an electrochemical analyzer (HypairFeNO Medisoft Exp’air, 2010) according to ATS-ERS recommendations for the online measurement of FeNO in adults. FeNO measurement was performed according to guidelines. The measurement range was 0–600 ppb. Exhaled NO (FeNO) was measured using a restricted breathing technique that employed expiratory resistance and positive mouth pressure to close the veil and exclude nasal NO and a constant expiratory flow of 50 mL/s. Repeated exhalations were performed until three plateaus agreed within 5% of the difference between observations [16].

### 2.6. Data Analysis

All analyses were conducted with SPSS-25 (SPSS, Chicago, IL, USA). We verified the distribution of the continuous variables under study using the one-sample Kolmogorov–Smirnov test and we verified that almost all of them had a normal distribution. Continuous parametric variables were expressed as m (mean) ± sd (standard deviation). Continuous non-parametric variables were expressed as median (interquartile Range (IQ) 25, 75). The dichotomous or non-continuous variables were expressed as %. For continuous parameters with normal distribution, a Student’s *t*-test was performed for paired samples for the comparison between time T0 and time T1 of the general population and subgroups. A Student’s *t*-test for independent samples was performed to compare the variables between subgroups both at time T0 and at time T1.

For continuous variables with non-parametric distribution, the comparison for independent samples was performed by the Mann–Whitney U test and the comparison for paired samples by the Wilcoxon test.

The chi-square test was used for the comparison of the discontinuous variables.

By means of binomial logistic regression tests, we verified which parameters were significantly predictive of ΔFeNO 50 < 0 and of LTRA therapy. To correctly classify the ΔFeNO 50 < 0 Group with the ΔFeNO 50 ≥ 0 Group, linear canonical discriminant analysis was used to create a model that optimizes the between-sample classes and within-sample class distances. The cross-validated accuracy percentage (CVA, %) was calculated.

A significance value of *p* < 0.050 was assumed for all analyses.

## 3. Results

### 3.1. Demographics and Baseline Characteristics

Our population had a mean age of 59.08 ± 11.09 years and 50% were female. At time T0, 16.7% of patients started omalizumab therapy, 63.9% initiated therapy with mepolizumab and 19.4% started with benralizumab. A total of 41.7% of patients who were on LTRA therapy at T0 time were also on T1 time.

Mean body mass index (BMI) was 10.72 ± 14.38. Mean time to asthma in years was 27.52 ± 18.11 with a median of 52 years (11–56). All patients were atopic, had an eosinophilic phenotype with a mean of 831.57 ± 503.59 and had FeNO 50 values greater than 25 with a mean of 73.40 ± 76.41. The mean ΔFeNO between time T0 and T1 time was −4.73 ± 48.92. The mean IgE tot value was 954.61 ± 3410.49 (Table 1).

At time T0, in the previous year, the number of antibiotic courses was 2.68 ± 1.53, the number of OCS courses was 6.18 ± 2.94, the number of bronchial exacerbations was 5.62 ± 3.65, the number of hospitalizations was 0.94 ± 0.91 and the number of unplanned visits to specialist was 4.63 ± 1.97.

At T1 time, 6 months after starting biological therapy, the number of antibiotic courses was 0.25 ± 0.47, the number of OCS courses was 0.62 ± 1.60, the number of bronchial exacerbations was 0.48 ± 0.78, the number of hospitalizations was 0.26 ± 0.55 and the number of unplanned visits to specialist was 1.82 ± 1.38.

### 3.2. Comparison between Group

#### 3.2.1. Comparison within the Entire Population between T0 and T1

As can be seen from Table 2, after 6 months from the start of biological therapy, the need for reliever therapy was significantly lower (63.8% vs. 21.2%; *p* = 0.007) and the eosinophil values significantly decreased (680.00 (500.00–959.00) vs. 130.00 (97.50–447.50); *p* = 0.004). Reduction in blood eosinophilia remained significant when calculated among patients under therapy with mepolizumab (1136 ± 812.90 vs. 178.00 ± 127.94; *p* = 0.040) and benralizumab (1117.60 ± 622.10 vs. 90.00 ± 57.00; *p* = 0.022), but not omalizumab (655.00 ± 224.87; *p* = ns).

At time T1 there was also an improvement in both FEV 1 (T0 vs. T1: 73.37 ± 21.72 vs. 85.67 ± 16.07; *p* = 0.000) and FVC (T0 vs. T1: 94.66 ± 17.91 vs. 96.51 ± 16.13; *p* = 0.000).

We made a sub-analysis comparing the collected data of the 6 months pre-biological therapy with the data of the 6 months post-introduction of the therapy. The results show a significant improvement in all the clinical outcome parameters considered.

Six months before starting biological therapy, the number of antibiotic courses was 0.77 ± 1.04, the number of OCS courses was 1.41 ± 1.82, the number of bronchial exacerbations was 2.73 ± 1.70, the number of hospitalizations was 0.33 ± 0.53 and the number of unplanned visits to specialist was 3.30 ± 1.47.

Compared with the previous 6 months, 6 months after starting biological therapy, the number of antibiotic courses was 0.25 ± 0.47 (*p* = 0.002), the number of OCS courses was 0.62 ± 1.60 (*p* = 0.001), the number of bronchial exacerbations was 0.48 ± 0.78 (*p* = 0.000), the number of hospitalizations was 0.26 ± 0.55 (*p* = 0.005) and the number of unplanned visits to specialist was 1.82 ± 1.38 (*p* = 0.000), which was significantly reduced.

#### 3.2.2. Comparison of Subgroups Based on LTRA Therapy (LTRA–No LTRA)

Of the 36 patients, 46.7% (*n* = 15) were taking LTRA therapy at baseline (T0). The mean age was similar between those who were on LTRA therapy (59.67 ± 8.88) and those who were not (58.67 ± 12.64). The percentage of females was also similar between the two subpopulations (53.3 vs. 47.6). All other parameters studied at baseline were similar between the two subpopulations (Supplementary Table S1).

**Figure 1:**
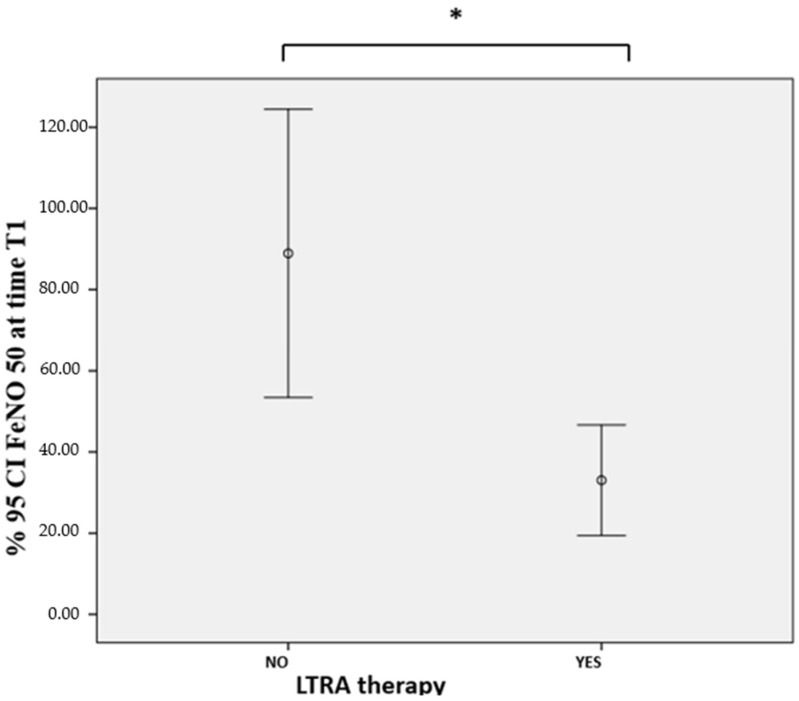
FeNO 50 at time T1: comparison based on LTRA therapy. FeNO: exhaled nitric oxide; LTRA: leukotriene receptor antagonist. *: statistically significant difference; LTRA therapy vs. no LTRA therapy: 30.83 ± 24.30 vs. 88.93 ± 77.96; *p* = 0.009.

As can be seen in Table 3, patients with LTRA therapy presented, at time T1 (6 months after the introduction of biological therapy), significantly lower values of FeNO 50 (30.83 ± 24.30 vs. 88.93 ± 77.96; *p* = 0.009) (Figure 1) and unplanned visits to specialists (1.26 ± 0.07 vs. 2.40 ± 1.17; *p* = 0.003).

#### 3.2.3. Comparison of Subgroups Based on the Parameter ΔFeNO (ΔFeNO < 0 Group and ΔFeNO 50 ≥ 0 Group)

Overall, the subpopulation with ΔFeNO < 0 (*n* = 12) and that with ΔFeNO 50 ≥ 0 (*n* = 24) presented similar mean age (59.25 ± 11.31 vs. 59.00 ± 11.23) and anthropometric, demographic, comorbid and lifestyle characteristics at the baseline (Supplementary Table S2).

Among the subjects who presented an improvement in FeNO (Group 1; ΔFeNO < 0) compared to the others (Group 0; ΔFeNO ≥ 0), there were a significantly greater number of patients on LTRA therapy (66.7% vs. 29.2%; *p* = 0.037).

Group 1 compared to Group 0 also performed statistically significantly fewer cycles of antibiotic therapy at time T0 (2.00 ± 1.33 vs. 4.14 ± 1.34; *p* = 0.005). Finally, Group 1 compared to Group 0 showed less unplanned specialist visits at T1 (0.92 ± 0.85 vs. 2.44 ± 0.96; *p* = 0.000) (Table 4).

Overall, the other parameters considered by the study were similar between the two groups at times T0 and T1.

Between the times T0 and T1 in Group 1 (ΔFeNO < 0), improvements were recorded in FEV 1 (83.83 ± 13.08 vs. 68.00 ± 10.81), asthma control (ACT: 20.91 ± 3.65 vs. 14.54 ± 5.55; ACQ: 0.72 ± 0.48 vs. 1.87 ± 0.93), need of inhaled reliever medication (5% vs. 66.7%) and average dose of oral corticosteroid use (1.15 ± 3.37 vs. 9.22 ± 9.31). 

Between the times T0 and T1 in Group 0 (ΔFeNO 50 ≥ 0) improvements were recorded in FEV 1 (84.36 ± 21.21 vs. 74.15 ± 26, 01) and asthma control test (ACT: 20.62 ± 4.59 vs. 14.34 ± 4.89).

### 3.3. Prediction Analysis on LTRA Therapy Efficacy and FeNO Reduction

By means of binomial logistic regression tests, we verified that the presence of therapy with LTRA (ODD 4.85 [1.09–21.51]; *p* = 0.037) and low number of unplanned specialist visits (ODD = 0.10 [0.02–0.50]; *p* = 0.005) were significant predictors of FeNO 50 reduction 6 months after the introduction of biological therapy (Table 5).

Using the binomial logistic regression test it was also verified that low FeNO values at time T1 (ODD = 0.97 (0.93–0.99); *p* = 0.024) and a low number of unscheduled specialist visits (ODD = 0.331 [0.144–0.761]; *p* = 0.009) were statistically significant predictors of LTRA therapy. We carried out the multivariate binomial logistic regression by introducing in the predictive model the parameters that were significant in the univariate model (low FeNO values at time T1; low number of unscheduled specialist visits) and the main confounding factors (age, sex and BMI).

We have verified that both the low values of FeNO values at time T1 (ODD = 0.955 (0.919–0.993); *p* = 0.021) and a low number of unscheduled specialist visits (ODD = 0.179 [0.048–0.763]; *p* = 0.011) maintain their prediction of LTRA therapy (Table 6).

**Figure 2:**
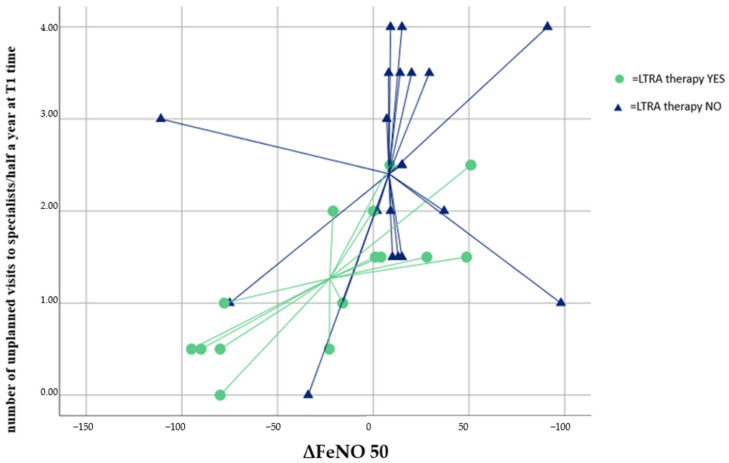
Discriminant Analysis. LTRA therapy discrimination with the number of unplanned specialist visits at time T1 and ΔFeNO 50. FeNO: exhaled nitric oxide; ΔFeNO50: FeNO 50 T0 − FeNO 50 T1. LTRA: leukotriene receptor antagonist. Cross-valid value 66.7%.

Finally, through the methodology of the Discriminant Analysis, it was verified that only the ΔFeNO parameters and the number of unscheduled specialist visits made it possible to correctly classify 66.7% of patients on LTRA therapy (Figure 2), with good model accuracy (AUC = 0.79 [0.64–0.93]; *p* = 0.003).

## 4. Discussion

Our study showed, to our knowledge, for the first time, that LTRA therapy, in a population of severe asthmatics with coexisting non-ABPA and non-CF bronchiectasis, may allow an increase in the beneficial effects of biological therapy, improving clinical control of asthma, reducing inflammation assessed by the FeNO biomarker and leading to a reduction in the number of unscheduled visits to specialists.

It is widely known from the literature that a reduction in FeNO improves asthma control [4] and that LTRA therapy was found to be beneficial for patients with aspirin-exacerbated respiratory disease, elderly asthma, smoking asthma, asthma associated with obesity and asthma comorbid with allergic rhinitis [20].

In our hypothesis, in a population of severe asthmatics with coexisting non-ASBA bronchiectasis and non-CF, the LTRA therapy would allow the reduction of FeNO with consequent better clinical control by acting both along the eosinophilic inflammatory pathway [4] and through the neutrophilic inflammatory pathway that is usually associated with exacerbations of bronchiectasis [5].

Although not always confirmed [21], FeNO was found to be increased even in the presence of bronchiectasis. In 1995, it was shown that elevated levels of exhaled NO in BE were correlated with disease severity [9]. There is an inducible isoform of NO synthase (iNOS) which is induced by proinflammatory cytokines and endotoxin in a variety of cells, including macrophages and airway epithelial cells [15]. It is possible that activated macrophages and neutrophils may express iNOS in bronchiectasis airways, since these cell types have been shown to express iNOS in response to cytokines [9]. The levels of FeNO in bronchiectasis can therefore probably be related to the inflammatory state of the same. In fact, the mean value of FeNO in patients with stable bronchiectasis was 8.8 ± 1.5 part per billion (ppb). This value was increased if, in addition to bronchiectasis, the patients presented asthma (48.0 ± 6.4 ppb) or COPD (31.0 ± 4.3) [22]. Asthmatic patients with fractional exhaled nitric oxide (FeNO) 50 > 22.5 parts per billion (ppb) were more likely to have coexisting bronchiectasis [7]. It is therefore not surprising that in our population made up of asthmatic atopic subjects not in perfect clinical control and also with bronchiectasis, the average values of FeNO were found to be high (73.40 ± 76.41). Reducing eosinophilic and neutrophilic activation through LTRA therapy could reduce NO production, leading to better control of asthma and bronchiectasis.

Furthermore, patients taking LTRA had a significant reduction in the likelihood of having unplanned specialist visits with an ODD = 0.33 [0.144–0.76]. The value of FeNO after 6 months from the start of biological therapy and the number of unscheduled specialist visits alone allowed the correct discrimination of 66.7% of patients with LTRA therapy. Therefore, if patients with severe asthma and bronchiectasis on biological therapy took LTRA therapy, they were more likely to reduce the inflammatory biomarker FeNO and reduce the number of extra specialist visits, with potential cost savings.

As we already started to discuss, a possible explanation of these data once again is to be found in the mechanism of action of LTRA, not only on the inflammatory eosinophilic axis [4] but also on the neutrophilic axis [23].

The cysLT/cysLTR1 axis is therefore involved in the control of neutrophilic inflammation. Few studies to evaluate this inflammatory axis have been conducted in patients with bronchiectasis. The absence of randomized trials led to a review of “Leukotriene receptor antagonists for non-CF bronchiectasis” to conclude that we cannot rule out an effect of leukotriene antagonists in the therapeutic management of bronchiectasis [23]. Moreover, the action of the cysLT/cysLTR1 axis is also expressed at the level of neutrophils, determining increase Ca^2+^ inflow, production of nitric oxide and ROS. In addition to the anti-inflammatory activity obtained through the cysLTR1 blocker, LTRA also has anti-inflammatory properties, primarily against neutrophils and monocytes/macrophages, which are independent of cysLTR1 antagonism. It has been described that on neutrophils and macrophages they are able both to reduce the activity of 5-lipoxygenase and to be P2Y receptor antagonists. At the level of neutrophils, they are able to determine inhibition of cyclic nucleotide phosphodiesterases [10]. So, improving control of the neutrophilic as well as eosinophilic pathways through LTRA therapy can reduce the number of unscheduled visits in patients with severe asthma and bronchiectasis.

Another interesting result that emerges from our data is the confirmation, already seen in previous articles [8,24,25,26,27], that anti-Il-5 and anti-IGE biological therapy have been shown to be effective in reducing blood eosinophilia and in controlling severe asthma, regardless of whether patients also had bronchiectasis not due to CF or ABPA.

In general, in our population we observed a significant reduction in eosinophilia values at time T1 after 6 months of biological therapy (680.00 (500.00–959.00) vs. 130.00 (97.50–447.50); *p* = 0.004). More specifically, the reduction in blood eosinophilia in our population of asthmatics and bronchiectasis was significant in both patients taking mepolizumab and those taking bernalizumab, consistent with data from recent publications [24,25,26,27]. In our patients taking omalizumab we observed a reduction in eosinophilia, but without reaching statistical significance. Indeed, omalizumab in its mechanism of action, targeting immunoglobulin E (IgE), reduces exacerbations in patients with severe allergic asthma, regardless of the eosinophil count in the blood [28].

In addition to the overall reduction in blood eosinophilia, after 6 months from the start of biological therapy, the general picture of clinical control improved with an increase in FEV1 (73.37 ± 21.72 vs. 85.67 ± 16.07) and FVC (94.66 ± 17.91 vs. 96.51 ± 16.13) and a reduction in the need for inhaled reliever medications (63.8% vs. 21.2%). Our data are consistent with recent work on similar populations demonstrating the efficacy of mepolizumab in reducing inflammation and improving clinical control in a severe asthma population with coexisting bronchiectasis [8]. In a recent article, 12 patients were studied with the simultaneous presence of an eosinophilic and/or atopic phenotype and who also had radiographic diagnosis of bronchiectasis not due to CF or ABPA. The authors hypothesized that in this category of patients, biologics targeting type 2 inflammation were effective in clinical and inflammatory control, regardless of the specific type of drug, showing a general class effect of anti-type 2 drugs [29]. We found similar results in our study, although it is fair to point out that there were no patients with biological agents targeting Il-4 in our population.

The first limitation of our study is the short duration of the follow-up (6 months). The future aim of the study is to verify whether these data will be confirmed after at least 1 year of follow-up. An additional limitation of our study is the retrospective nature and the low number of patients enrolled. In our opinion, patients with bronchiectasis should also be included in large trials of severe asthma in the future.

In conclusion, the presence of LTRAs in therapy in a population of severe asthmatics with coexisting non-ASBA bronchiectasis and non-cystic fibrosis, acting simultaneously on the TH2 pathway and probably on the neutrophilic component of bronchiectasis, would allow a further amplification of the beneficial effects of biological therapy, leading to a reduction in the number of unplanned visits to specialists.

Future studies with a larger number of subjects will allow for improved therapy in this subgroup of patients.

## Figures and Tables

**Table 1:** Patients’ characteristics, asthmatic disease time, comorbidities related to asthma, radiological severity of bronchiectasis, type of biological therapy prescribed.

	Clinical Feature
Age, year m ± sd	59.08 ± 11.09
Female sex, %	50
BMI, kg/m^2^ m ± sd	24.86 ± 3.53
Smoker, %	
current	13.9
former	27.8
never	58.3
BSI score, m ± sd	7.09 ± 3.98
Bilateral bronchiectasis yes, %	84.6
Nodular pattern yes, %	13.9
Time of asthmatic disease, year m ± sd	27.52 ± 18.11
Family history of asthma yes, %	19.4
GERD yes, %	30.6
ASA sensibility yes, %	11.1
Allergic rhinitis yes, %	33.3
LTRA therapy yes, %	41.7
Biological therapy, %	
omalizumab	16.7
mepolizumab	63.9
bernalizumab	19.4

BMI: body mass index; BSI: bronchiectasis severity index; GERD: gastro-esophageal reflux disease; ASA sensibility: acetylsalicylic acid sensibility; LTRA: leukotriene receptor antagonist.

**Table 2:** Comparison of the entire population between the time T0 and T1.

	TO Time	T1 Time	*p* Value
FeNO 50, ppb m ± sd	73.40 ± 76.41	68.86 ± 69.74	0.595
EOS, cells/μL median (IQ 25–75)	680.00 (500.00–959.00)	130.00 (97.50–447.50)	0.004
IGE tot, KU/L median (IQ 25–75)	234.00 (119.00–534.50)	400.00 (215.00–700.00)	0.756
FEV1, m ± sd % predicted	73.37 ± 21.72	85.67 ± 16.07	0.000
FVC, m ± sd % predicted	94.66 ± 17.91	96.51 ± 16.13	0.000
RV, m ± sd % predicted	110.28 ± 28.08	112.00 ± 23.99	0.113
ACT, score m ± sd	14.41 ± 5.03	20.70 ± 4.31	0.219
ACQ, score m ± sd	1.95 ± 0.96	0.93 ± 0.69	0.431
Need of inhaled reliever medication, %	63.8	21.2	0.007
OCS, yes %	66.6	45.7	0.353
Average dose of OCS, mg m ± sd	13.59 ± 12.37	4.05 ± 7.04	0.550
FACED, score m ± sd	2.18 ± 1.16	1.42 ± 1.27	0.172
% Positive sputum	16.7	5.5	0.125
Mucolytic therapy, yes %	35	35	1.000
Adherence to therapy %	89	95	0.200
WBCs, cells/μL median (IQ 25–75)	7000.00 (6500.00–9725.00)	7100.00 (6590.00–10,420.00)	0.998

FeNO: exhaled nitric oxide; EOS: blood eosinophilia; FEV1: forced expiratory volume in the 1st second; FVC: forced vital capacity; IgE: immunoglobulin E; VR: residual volume; ACT: asthma control test; ACQ: asthma control questionnaire; OCS: oral corticosteroid; WBCs: white blood cells; LTRA: leukotriene receptor antagonist.

**Table 3:** Comparison based on taking LTRA therapy 6 months after starting biological therapy.

T1 TIME			
	LTRA Yes (*n* = 15)	LTRA No (*n* = 24)	*p* Value
FeNO 50, ppb m ± sd	30.83 ± 24.30	88.93 ± 77.96	0.009
EOS, cells/μL median (IQ 25–75)	150.00 (125.00 380.00)	100.00 (65.00–265.00)	0.201
IgE, KU/L median (IQ 25–75)	555.00 (400.00–700.00)	215.00 (132.00–324.00)	0.067
FEV1, m ± sd % predicted	80.34 ± 19.20	87.58 ± 18.59	0.305
FVC, m ± sd % predicted	94.97 ± 23.91	98.64 ± 27.07	0.707
ACT, score m ± sd	20.13 ± 4.06	22.00 ± 4.26	0.193
ACQ, score m ± sd	1.12 ± 0.88	0.94 ± 0.81	0.663
Need of inhaled reliever medication, %	20.0	20.0	0.709
OCS yes, %	40.0	47.6	0.456
Average dose of OCS, mg m ± sd	3.55 ± 4.99	4.19 ± 8.30	0.503
Adherence to therapy, %	90.0	100.0	0.500
WBCs, cells/μL median (IQ 25–75)	8950.00 (7000.00–10,900.00	7100.00(6590–9275)	0.555
OCS course, n m ± sd	0.53 ± 1.34	0.69 ± 1.79	
Bronchial exacerbation/6 months n m ± sd	0.41 ± 0,77	0.53 ± 0.80	0.639
antibiotic courses/6 months n m ± sd	0.20 ± 0.41	0.28 ± 0.51	0.584
Number of hospitalizations/6 months n m ± sd	0.10 ± 0.20	0.50 ± 0.70	0.052
Unplanned visits to specialists/6 months n m ± sd	1.26 ± 0.07	2.40 ± 1.17	0.003

FeNO: exhaled nitric oxide; EOS: blood eosinophilia; FEV1: forced expiratory volume in the 1st second; FVC: forced vital capacity; IgE: immunoglobulin E; VR: residual volume; ACT: asthma control test; ACQ: asthma control questionnaire; OCS: oral corticosteroid; WBCs: white blood cells; LTRA: leukotriene receptor antagonist.

**Table 4:** Comparison between Groups 1 and 0, respectively, at times T0 and T1.

Parameters	T0 Time			T1 Time		
	Group1	Group 0	*p*	Group 1	Group 0	*p*
* FeNO 50, ppb m ± sd	125.32 ± 99.57	45.28 ± 36.55	0.019	71.36 ± 96.08	64.37 ± 52.07	0.781
* EOS, cells/μL median (IQ 25–75)	700.00 (555.00–1084.00)	620.00(500.00–898.00)	0.379	130.00 (110.00–150.00)	400.00 (90.00–700.00)	0.548
IgE, KU/L median (IQ 25–75)	300.00 (118.00–899.00)	213.00 (147.50–384.50)	0.450	500.00 (400.00–650.00)	260.00–(173.50–631.50)	0.344
* ^#^ FEV1, m ± sd % predicted	68.00 ± 10.81	74.15 ± 26.01	0.504	83.83 ± 13.08	84.36 ± 21.21	0.934
FVC, m ± sd % predicted	81.50 ± 16.26	92.20 ± 24.79	0.550	83.20 ± 16.68	103.10± 25.34	0.295
RV, m ± sd % predicted	118.75 ± 35.18	112.82 ± 30.89	0.789	94.66 ± 9.07	119.42 ± 24.93	0.051
* ^#^ ACT, score m ± sd	14.54 ± 5.55	14.34 ± 4.89	0.921	22.41 ± 3.23	20.62 ± 4.59	0.236
* ACQ, score m ± sd	1.87 ± 0.93	2.14 ± 1.21	0.753	0.72 ± 0.48	1.31 ± 0.99	0.137
Positive sputum, %	33.8	8.3	0.080	8.3	4.2	0.562
* Need of inhaled reliever medication, %	66.7	60.7	0.570	5	30	0.172
LTRA Therapy, yes %	66.7	29.2	0.037	66.7	29.2	0.037
OCS, yes %	63.6	70.8	0.479	27.3	54.2	
* Avarage dose of OCS	9.22 ± 9.31	15.56 ± 13.26	0.154	1.15 ± 3.37	5.12 ± 7.99	0.183
OCS course, n m ± sd	6.14 ± 3.07	6.22 ± 3.03	0.960	0.50 ± 1.73	0.69 ± 1.57	0.746
Bronchial of exacerbation/n m ± sd	4.54 ± 2.16	6.12 ± 4.11	0.241	0.34 ± 0.61	0.55 ± 0.86	0.416
Mucolytic therapy, yes %	50	20	0.175	50	20	0.175
Adherence therapy %	81.8	91.7	0.372	100	90	0.500
WBCs, cells/μL median (IQ 25–75)	7000 (6500–8500)	10,400 (7700.00–11,100.00)	0.069	7000.00 (6785.00–7050.00)	9250.00 (7160.00–10,660.00)	0.256
* Antibiotic courses, n m ± sd	2.00 ± 1.33	4.14 ± 1.34	0.005	0.12 ± 0.22	0.31 ± 0.55	0.266
Hospitalizations n m ± sd	0.88 ± 0.78	1.00 ± 1.05	0.796	0.10 ± 0.31	0.40 ± 0.69	0.232
Unplanned visits to specialists n m ± sd	4.66 ± 2.00	4.60 ± 2.06	0.944	0.92 ± 0.85	2.44 ± 0.96	0.000

Group1: ΔFeNO 50 < 0. Group 0: ΔFeNO 50 ≥ 0. FeNO: exhaled nitric oxide; EOS: blood eosinophilia; FEV1: forced expiratory volume in the 1st second; FVC: forced vital capacity; VR: residual volume; ACT: asthma control test; ACQ: asthma control questionnaire; OCS: oral corticosteroid; WBCs: white blood cells; LTRA: leukotriene receptor antagonist. * INTRA-GROUP ANALYSIS GROUP 1: Significantly different parameters (*p* value < 0.050) within Group 1 (ΔFeNO 50 < 0) between times T0 and T1. ^#^ INTRA-GROUP ANALYSIS GROUP 0: Significantly different parameters (*p* value < 0.050) within Group 1 (ΔFeNO 50 ≥ 0) between times T0 and T1.

**Table 5:** Prediction of FeNO reduction.

	ODD	CI	*p*
Biological therapy	1.26	0.39–4.03	0.695
LTRA therapy	4.85	1.09–21.51	0.037
Unplanned visits to specialists at T1 time	0.10	0.02–0.50	0.005
ΔACT	1.01	0.91–1.01	0.762
Age	1.01	0.94–1.06	0.948
Sex	1.65	0.40–6.71	0.481

ACT: asthma control test. ΔACT = ACT T1 − ACT T0. FeNO: exhaled nitric oxide. LTRA: leukotriene receptor antagonist. Probability of having a reduction in FeNO 50 in relation to the type of biological therapy. to the main confounding factors (age and sex) and to the parameters that were statistically significant for *p* < 0.050.

**Table 6:** Prediction of LTRA therapy.

	Univariate Regression Analysis			Multivariate Regression Analysis		
	ODD	IC 95%	*p* Value	ODD	IC 95%	*p* Value
Age, y	1.008	0.949–1.072	0.787	1.049	0.947–1.163	0.360
Sex, female sex	1.257	0.333–4.742	0.735	0.484	0.034–4.994	0.484
BMI, kg/m^2^	1.176	0.957–1.445	0.123	0.136	0.909–2.008	1.351
FeNO 50 T1, ppb	0.97	0.93–0.99	0.024	0.955	0.919–0.993	0.021
Unplanned visits to specialists T1/6 months	0.331	0.144–0.761	0.009	0.179	0.048–0.673	0.011

Probability of LTRA therapy. ACT: asthma control test. ΔACT = ACT T1 − ACT T0. FeNO: exhaled nitric oxide. LTRA: leukotriene receptor antagonist. Statistical significance was assumed for *p* < 0.050.

## Data Availability

Not applicable.

## Supplementary Materials

The following supporting information can be downloaded at: https://www.mdpi.com/article/10.3390/jcm11164702/s1, Table S1: Baseline comparison based on LTRA therapy intake; Table S2: Baseline comparison based on ΔFeNO 50 of Patients’ characteristics, comorbidities related to asthma, radiological severity of bronchiectasis, type of biological therapy prescribed.

## Abbreviations

cysLT/cysLTR1	cysteinyl leukotriene/cysteinyl leukotriene receptor 1
LTRA	leukotriene receptor antagonist
non-CF	non-cystic fibrosis
non-ABPA	non-allergic bronchopulmonary aspergillosis
ACT	asthma control test
ACQ	asthma control questionnaire
IgE	immunoglobulin E
FeNO 50	fractional exhaled nitric oxide 50
ΔFeNO	FeNO 50 T1 − FeNO 50 T0
TH2	T helper type 2
ppb	parts per billion
iNOS	inducible nitric oxide synthase
NO	nitric oxide
SA-centers	severe asthma centers
CT	computed tomography
GINA	Global Initiative for Asthma
T0	baseline
T1	after 6 months of biological therapy
SPT	skin prick test
F/V	flow/volume
FVC	forced vital capacity
FEV1	forced expiratory volume during the first second of the forced breath
RV	residual volume
ACT	Asthma Control Test
ACQ	Asthma Control Questionnaire
BSI	bronchiectasis severity index
FACED	FEV1, age, chronic pseudomonas aeruginosa bronchial infection colonization, radiological extension and dyspnea
WBC	white blood cell
EOS	eosinophilia
OCS	oral corticosteroid
CVA	cross-validated accuracy
BMI	body mass index
GERD	gastro-esophageal reflux disease
ASA sensibility	acetylsalicylic acid sensibility
