# Supplementary material for: Can Leukotriene Receptor Antagonist Therapy Improve the Control of Patients with Severe Asthma on Biological Therapy and Coexisting Bronchiectasis? A Pilot Study
Source: J Clin Med. 2022 Aug 11;11(16):4702. doi: 10.3390/jcm11164702 (PMC9410441; doi:10.3390/jcm11164702)
Supplement: Supplementary file 1 [file jcm-11-04702-s001.zip › jcm-1825841-supplementary.pdf]

Supplementary Table S1: Baseline comparison based on LTRA therapy intake

BASELINE			
	LTRA yes<br>(n=15)	LTRA no<br>(n=24)	P Value
Age , year m ± sd	59.67 ± 8.88	58.67 ± 12.64	0.782
Female sex, %	53.3	47.6	0.050
BMI, kg/m2 m ± sd	25.95 ± 2.66	24.08 ± 3.91	0.118
Smoker, %<br>current<br>former<br>never	 13.3<br>33.3<br>53,3	 14.3<br>23.8<br>61.9	0.818
BSI, score m ± sd	7.23 ± 2.94	6.00 ± 3.44	0.327
FACED, score m ± sd	2.37 ± 1.30	1.66 ± 0.57	0.247
Time of asthmatic disease year m ± ds	21.53 ± 13.42	31.80 ± 20.05	0.094
Allergic rhinitis yes, %	70.0	50.0	0.325
Nasal Polyposis yes, %	73.3	66.7	0.479
GERD yes, %	20.0	38.1	0.215
ASA sensibility yes, %	10.0	30.0	0.291
Biological Therapy, %<br>Omalizumab<br>Mepolizumab<br>Bernalizumab	 19.0<br>71.4<br>9.5	 20.0<br>53.3<br>26.7	0.369
FeNO 50, ppb m ± sd	53.59 ± 44.92	80.77 ± 90.09	0.243
EOS, cells/µL<br>median (IQ 25-75	600.00 (450.00-700.00)	711.00 (500.00 -1250.00)	0.109
IGE tot, KU/l<br>median (IQ 25-75	240.50 (116.00 – 800.00)	234.00 (132.00-384.500)	0.700
FEV1, m ± sd	71.23 ± 18.39	73.00 ± 25.72	0.834

% predicted			
FVC, m ± sd<br>% predicted	95.33 ± 13.05	77.00 ± 19.74	0.072
RV, m ± sd<br>% predicted	131.00 ± 43.84	111.42 ± 29.54	0.640
ACT, score m ± sd	13.57 ± 5.00	15.00 ± 5.09	0.423
ACQ, score m ± sd	1.76 ± 0.74	2.23 ± 1.45	0.552
Need of inhaled reliever medication, %	66.7	60.0	0.570
OCS yes, %	66.7	70.0	0.803
Avarage dose of OCS, mg/dl<br>m ± sd	13.30 ± 10.18	13.82 ± 14.24	0.913
FACED, score m ± sd	2.37 ± 1.30	1.66 ± 0.57	0.247
Positive Sputum %	30.0	14.3	0.170
Mucolytic therapy, yes %	20.0	40.0	0.175
Adherence to therapy, yes %	80.0	80.0	0.709
WBC , cells/µL<br>median (IQ 25-75)	7000.00 (6500.00 - 7350)	7100.00 (7000.00 - 10400)	0.603
OCS course, n m ± sd	5.87 ± 2.29	6.50 ± 3.62	0.688
Bronchial exacerbation<br>n m ± sd	4.57 ± 3.03	6.33 ± 3.92	0.145
* antibiotic course,<br>n m ± sd	2.66 ± 1.65	3.12 ± 1.80	0.596
Hospitalizations,<br>n m ± sd	0.77 ± 0.66	1.10 ± 1.11	0.447
Unplanned visits to specialists, n m ± sd	4.44 ± 1.87	4.80 ± 2.15	0.705

FACED score: FEV1% predicted. Age. Chronic colonization byPseudomonas aeruginosa. Radiological extent of the disease. Dyspnea; GERD: Gastro-esophageal reflux disease; ASA sensibility: acetylsalicylic acid

sensibility; FeNO: exhaled nitric oxide; EOS: blood eosinophilia; ; FEV1: Forced Expiratory Volume in the 1st second; FVC: Forced vital capacity; VR: residual volume; ACT: Asthma control test; ACQ: Asthma control questionnaire; OCS: oral corticosteroid ; WBC: White Blood Cells; LTRA: Leukotriene Receptor Antagonists

FACED score: FEV1% predicted. Age. Chronic colonization byPseudomonas aeruginosa. Radiological extent of the disease. Dyspnea; GERD: Gastro-esophageal reflux disease; ASA sensibility: acetylsalicylic acid sensibility; FeNO: exhaled nitric oxide; EOS: blood eosinophilia; ; FEV1: Forced Expiratory Volume in the 1st second; FVC: Forced vital capacity; VR: residual volume; ACT: Asthma control test; ACQ: Asthma control questionnaire; OCS: oral corticosteroid ; WBC: White Blood Cells; LTRA: Leukotriene Receptor Antagonists

Supplementary Table S2: Baseline comparison based on Δ FeNO50 of Patients' characteristics, comorbidities related to asthma, radiological severity of bronchiectasis, type of biological therapy prescribed

	**Group 1**<br>**(n=12)**	**Group 0**<br>**(n=24)**	**P Value**
Age<br>year m ± sd	59.25 ± 11.31	59.00 ± 11.23	0.951
Female sex, %	58.3	45.8	0.362
BMI , kg/m2 m ± sd	24.37 ± 3.60	25.11 ± 3.54	0.568
Smoker %<br>current<br>former<br>never	 16.7<br>33.3<br>50.0	 12.5<br>25.0<br>62.5	0.773
BSI m ± ds	5.75 ± 2.05	6.94 ± 3.58	0.286
Bilateral Bronchiectasis %	75	88.9	0.538

N. segments with bronchiectasis ,n m ± sd	8.00 ± 8.88	9.55 ± 5.85	0.798
FACED SCORE m ± sd	2.66 ± 0.51	1.60 ± 1.51	0.196
Time of asthmatic disease year m ± sd	28.17 ± 15.95	27.20 ± 19.43	0.876
Allergic rhinitis yes, %	50	50	0.672
GERD yes, %	30.6	29.2	0.544
ASA sensibility yes, %	20	20	0.709
Biological Therapy %			0.140
omalizumab	25.0	12.5	
mepolizumab	41.7	75.0	
bernalizumab	33.3	12.5	

Group1: Δ Feno 50 <0. Group 0: Δ Feno50 ≥ 0. FACED score: FEV1% predicted. Age. Chronic colonization byPseudomonas aeruginosa. Radiological extent of the disease. Dyspnea; GERD: Gastro-esophageal reflux disease; ASA sensibility: acetylsalicylic acid sensibility; FeNO: exhaled nitric oxide; Δ FeNO50: FeNO 50 T0 – FeNO 50 T1
